# Supplementary material for: Three Different Genetic Risk Scores Based on Fatty Liver Index, Magnetic Resonance Imaging and Lipidomic for a Nutrigenetic Personalized Management of NAFLD: The Fatty Liver in Obesity Study
Source: Diagnostics (Basel). 2021 Jun 13;11(6):1083. doi: 10.3390/diagnostics11061083 (PMC8231822; doi:10.3390/diagnostics11061083)
Supplement: Supplementary file 1 [file diagnostics-11-01083-s001.zip › diagnostics-1222244-supplementary.pdf]

Supplementary material

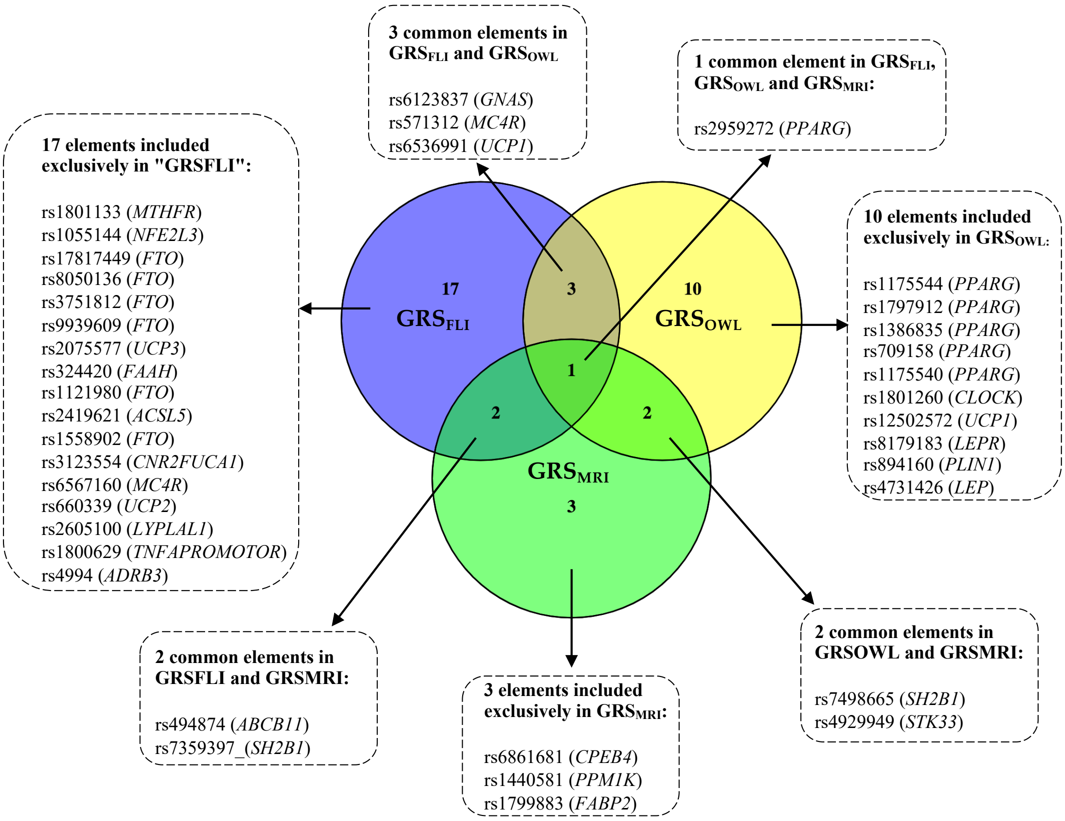

Figure S1. Venn diagram showing the number of SNPs associated with each NAFLD non-invasive diagnostic methods. GRS, Genetic Risk Score; MRI, Magnetic Resonance Imaging; FLI, Fatty Liver Index; OWL, OWLiver®-test.
